# An Exploratory Survey of Knowledge, Attitudes, and Behaviors Toward Cosmetic Products †

**DOI:** 10.3390/toxics14010068

**Published:** 2026-01-12

**Authors:** Selma Yazar, Burçin Şeyda Çorba, Hatice Ertuğrul, Ayşe Nurşen Başaran

**Affiliations:** 1 Department of Pharmaceutical Toxicology, Faculty of Pharmacy, Istanbul Yeni Yuzyil University, 34010 Istanbul, Türkiye; selma.yazar@yeniyuzyil.edu.tr (S.Y.); ertugrulhatice1568@gmail.com (H.E.); 2 Department of Statistics, Ondokuz Mayıs University, 55200 Samsun, Türkiye; burcinseyda.corba@omu.edu.tr; 3 Department of Pharmaceutical Toxicology, Faculty of Pharmacy, Başkent University, 06800 Ankara, Türkiye; anbasaran@baskent.edu.tr

**Keywords:** cosmetics, toxicity, cosmetovigilance, Türkiye, public health

## Abstract

Objective: Cosmetic products are widely used, yet public awareness of their potential health risks and of cosmetovigilance remains limited. Given that studies increasingly highlight chemical exposure associated with cosmetics, this study aimed to assess public knowledge, attitudes, and behaviours regarding cosmetic use, toxicity, and cosmetovigilance in Türkiye. Methods: A cross-sectional study was conducted among the general population living in Türkiye, consisting of 700 people between January and May 2024. The study was conducted using a Google survey form. Results: Among 700 participants, 91.6% reported regular cosmetic use and 47.6% experienced at least one adverse effect, most commonly redness, itching, and burning. Adverse effects were more frequently associated with products purchased from shopping malls/cosmetic stores. Education level was significantly linked to awareness of cosmetovigilance and product preferences, with university graduates showing higher awareness and favoring both local and international brands. Conclusion: The study revealed that although cosmetic use is common in Türkiye, awareness of cosmetovigilance remains low, even among well-educated consumers. Many participants reported adverse effects but did not seek professional consultation, indicating gaps in safety practices and reporting. Strengthening public awareness and establishing effective cosmetovigilance systems are essential to ensure safer cosmetic use and protect public health.

## 1. Introduction

Cosmetics are defined as “substances applied, poured, sprinkled or sprayed, placed or otherwise applied to the human body to cleanse, beautify, enhance attractiveness, or alter appearance” according to their intended use. They are widely used across all socioeconomic classes, and evidence suggests that the vast majority of users tend to prioritize the immediate positive effects on their appearance rather than the potential long-term consequences for their health [1,2]. In Türkiye, the cosmetics sector has grown in parallel with rising living standards, driven by increased awareness of personal care among both men and women and broader participation in professional and social life. Today, many domestic and international cosmetic companies are active in Turkey, reflecting the sector’s expansion and competitiveness [3]. In addition, many consumers hold the perception that cosmetic products are entirely risk-free and harmless to human health. However, although cosmetic products are required to comply with general safety requirements, the absence of mandatory product-specific testing remains a significant limitation. In particular, under FDA regulations—which do not require standardized pre-market evaluation—some formulations may reach consumers without a comprehensive safety assessment [2,4,5]. Although cosmetic products are increasingly regulated, especially in Europe following the full implementation of the EU Cosmetic Regulation in 2013, important gaps in consumer and environmental protection persist. Safety assessments often rely on historical data and older animal studies, while efforts to implement new approach methodologies and improve evaluation processes are still ongoing. Moreover, both illegal products and legally marketed products with unrecognized risks may be perceived by consumers as inherently safe. Adverse events related to cosmetic use are frequently underreported, limiting the ability of regulatory authorities to identify potential risks and implement timely corrective or preventive measures [6,7]. With the increasing use of cosmetic products, many individuals are unknowingly exposed to various side effects and toxic effects, thereby putting their health at risk [2,8]. Regulatory authorities aim to address these risks through strengthened safety assessments, post-marketing surveillance systems, and cosmetovigilance reporting requirements.

Epidemiological and toxicological evidence suggests that the adverse health effects of cosmetic products are primarily dermatological in nature, encompassing conditions such as contact dermatitis (both allergic and irritant), phototoxic and photoallergic responses, contact urticaria, and alterations in skin pigmentation (hyperpigmentation or hypopigmentation). Some studies have also suggested potential systemic health effects, such as acute poisoning or respiratory symptoms, although evidence remains limited [9,10,11]. The toxicological risks associated with cosmetic products appear to increase in parallel with their widespread use; however, user awareness regarding these risks has not shown a proportional rise. To safeguard public health, several countries have implemented cosmetovigilance systems—formal frameworks for the reporting and monitoring of adverse events related to cosmetic products [12]. Despite this, many developing countries still lack adequate infrastructures for adverse event reporting [13]. Strengthening cosmetovigilance practices and implementing awareness programs concerning the safe use of cosmetic products are therefore considered essential strategies to mitigate toxic effects and enhance consumer safety [2,3,4,5,6,7,8,9,10,11,12,13,14]. In Türkiye, previous cosmetovigilance research has been limited to a single study conducted only among female nurses [15]. In contrast, our survey includes the general population and additionally investigates both cosmetovigilance awareness and perceived toxic effects, thereby addressing a significant gap in the national literature. This study addresses cosmetic safety and consumer exposure from a public health and regulatory toxicology perspective, thereby fitting within the scope of the section. Therefore, our survey includes the general population and additionally investigates both cosmetovigilance awareness and perceived toxic effects, thereby addressing a significant gap in the national literature.

## 2. Materials and Methods

### 2.1. Study Design and Settings

This study was approved by the Ethics Committee at Istanbul Yeni Yuzyil University Science, Social and Non-Interventional Health Sciences Research Ethics Committee (Approval no: 2024/01-1165) and informed consent was obtained from all individual participants included in the study.

This study was designed as a cross-sectional survey conducted in Türkiye between January and May 2024. Participation was entirely voluntary, and electronic informed consent was obtained from all participants prior to data collection. Participants were informed of their right to withdraw from the study at any time without consequences. No personally identifiable information was collected, and all responses were anonymized.

### 2.2. Participants

Participants were recruited using a convenience sampling approach through online dissemination of the survey link via social media platforms. Individuals who accessed the survey link were informed about the study objectives and eligibility criteria before participation.

#### 2.2.1. Inclusion Criteria

Participants were eligible for inclusion if they:

- Were aged between 15 and 65 years,
- Resided in Türkiye,
- Were cosmetic product consumers,
- Completed the questionnaire in full.

#### 2.2.2. Exclusion Criteria

Participants were excluded if they:

- Were outside the predefined age range,
- Did not provide informed consent,
- Submitted incomplete or duplicate questionnaires.

### 2.3. Data Collection Tool

Data were collected using a self-developed questionnaire created in Google Forms (Google LLC, Mountain View, CA, USA), available in both Turkish and English. The survey link was distributed and shared through social media platforms. Data were collected using a self-developed questionnaire consisting of 25 items. The questionnaire was developed based on a review of relevant literature, and the items were adapted and combined to create a new instrument aligned with the objectives of the present study. The questionnaire included items assessing demographic characteristics, cosmetic product use, knowledge about cosmetic ingredients and their potential toxic effects, and awareness, attitudes, and behaviors related to cosmetovigilance. The questionnaire was not formally divided into separate sections; however, all items were designed to collectively evaluate knowledge, attitudes, and behaviors regarding cosmetic safety. All questions were categorical in nature and consisted of multiple-choice or yes/no response options. The selection of items was guided by previously published studies and aimed to comprehensively assess consumer awareness and practices related to cosmetic safety and cosmetovigilance. The country options included in the questionnaire were selected based on the cosmetic products most commonly available on the Turkish market. These options reflect the countries of origin most frequently encountered by consumers during routine cosmetic purchases. One item in the questionnaire addressed the statement “Cosmetic products are first tested on animals.” This item was deliberately included to assess misinformation and misconceptions among consumers regarding cosmetic safety regulations, particularly the ban on animal testing under European Union legislation. The inclusion of this item was methodologically justified as an indicator of public awareness gaps relevant to cosmetovigilance and consumer education.

### 2.4. Pilot Study

Prior to the main study, the questionnaire was administered to a pilot sample of 100 participants to evaluate potential technological issues and limitations. The results obtained from the pilot survey were not included in the final data analysis.

### 2.5. Statistical Analysis

A formal sample size calculation was not performed, as the study was based on voluntary participation in an online survey. The research data was loaded into a computer environment and analyzed using IBM SPSS 22 (IBM Statistical Package for Social Sciences, Corp., Armonk, NY, USA). Descriptive statistics for categorical variables were presented as frequencies and percentages. Cross-tabulations were used to compare categorical variables, and the “Pearson Chi-Square Test,” and “Yates Continuity Correction,” were applied. For significant results, subgroup tests were interpreted using the “Bonferroni Correction.” For categorical variables, correlation coefficient, “Contengency Coefficent” or “Phi/Cramer’s V” were applied. The normality distribution of numerical variables was assessed using the “Kolmogorov–Smirnov” or “Shapiro–Wilk” tests. Descriptive statistics of numerical variables were presented as mean ± standard deviation and median (minimum-maximum value). The level of statistical significance was accepted as *p* < 0.05.

## 3. Results

### 3.1. Demographic Characteristics and Cosmetics Use Habits

The demographic data of the participants are presented in Table 1. Among the 700 participants, 91.6% reported using cosmetics. Most participants purchased cosmetic products from shopping malls or cosmetic stores (*n* = 466), followed by pharmacies (*n* = 377) and online shopping platforms (*n* = 309). Moisturizers (*n* = 579) and facial care products (*n* = 513) were the most commonly used product categories. Furthermore, 595 participants stated that they regularly checked shelf life, expiration date, and product ingredients before purchasing. In terms of brand preference, Turkish cosmetic products were chosen most frequently (60.4%), followed by French brands (41.9%). For product-related information, the majority relied on the internet (*n* = 514), while 307 participants consulted pharmacists and 218 asked store staff. Daily cosmetic product use was reported by 550 participants, while 149 used them several times per week. None reported using cosmetics only when needed. Most participants (62.7%) stated that their primary reason for using cosmetics was protection.

### 3.2. Adverse Effects and Safety of Cosmetic Products

Information regarding cosmetic toxicity and safety is presented in Table 2. A total of 333 participants reported experiencing adverse effects from cosmetic products. The most frequently reported adverse effects were skin redness (*n* = 219) and itchy skin (*n* = 168). Following the experience of adverse effects, 383 participants discontinued product use, while 411 switched to a different brand, and only 18 continued using the product without any changes. Regarding medical consultation, 85 participants consulted only a physician, 37 consulted only a pharmacist, and 21 consulted both.

### 3.3. Awareness of Cosmetovigilance and Associated Factors

As presented in Table 3, although women reported knowing the definition of cosmetovigilance more frequently than men (105 vs. 11), no statistically significant correlation was found between gender and knowledge of the term (*p* = 0.078). A significant correlation was observed between purchasing cosmetics from pharmacies and awareness of cosmetovigilance (13.4%, *p* < 0.001). Among those who knew the term, 69% reported buying cosmetics from pharmacies, while 31% did not.

As presented in Table 4, among 641 participants who reported using cosmetic products, awareness of the term cosmetovigilance was higher among associate degree/university graduates (20.9%) compared to other education levels. A significant correlation of 15.4% was found between education level and knowledge of the term (*p* = 0.001). The belief that “cosmetic products are beneficial” was more common among associate degree/university graduates (74.5%), with a significant correlation of 17.4% (*p* = 0.003).

As shown in Table 5, adverse effects related to cosmetic products were significantly associated with the place of purchase and awareness of the term ‘cosmetovigilance’. Among participants reporting adverse effects, 62.2% purchased their products from pharmacies. Those who bought products from shopping malls or cosmetic stores reported adverse effects more frequently (72.1%) than those who did not (61.6%), showing a significant association (*p* = 0.039). Furthermore, participants aware of the term ‘cosmetovigilance’ were more likely to report adverse effects, with a significant correlation of 7.5% (*p* = 0.046).

## 4. Discussion

The present study provides important insights into consumer habits and awareness regarding the use of cosmetic products. A significant portion of participants (91.6%) reported using cosmetics regularly, with 46% using them daily. In the present study, 47.6% of participants—predominantly women—reported experiencing adverse effects; however, 72.4% of them did not seek medical consultation. Similar findings have been documented in studies conducted in various countries [14,15,16,17,18,19]. Across Europe, this trend is also evident, with reports indicating that adverse effects from cosmetic products are often underestimated, leading consumers to attempt self-treatment [20]. It has been emphasized that consumers should seek advice from healthcare professionals, particularly pharmacists, when experiencing cosmetic-related adverse effects. Such reporting is crucial not only for ensuring individual safety but also for strengthening cosmetovigilance systems [21]. Moreover, responsibility for the safe use of cosmetic products is shared not only by consumers and healthcare professionals but also by manufacturers [22]. Skin-related symptoms such as redness (50%), itching (38.4%), and a burning sensation (31.3%) were the most frequently reported adverse effects. These outcomes are consistent with previous studies conducted in other countries [17,23,24,25], and are likely due to the direct contact of cosmetic products with the skin [26]. Similarly, studies focused on specific product categories like hair dyes also reported comparable reactions [19].

Regarding toxicological awareness, 85.1% of participants believed that cosmetics could accumulate in the body, but only 28.6% were aware of the potential toxicity of nanoparticles. This knowledge gap may be related to participants’ reliance on internet sources instead of seeking professional guidance from pharmacists. Although participants had a relatively high level of education, the limited availability of reliable information in the Turkish cosmetics sector may contribute to inadequate consumer knowledge [21,27]. These findings highlight the importance of pharmacist consultation in the safe use of cosmetic products [26].

In terms of consumer behavior, 81.3% of participants reported researching cosmetic products online, and 44.1% indicated purchasing them through the internet. Similar trends have been documented in India and Türkiye [12,28], suggesting that the use of digital platforms for cosmetic-related decisions is becoming a global trend. While the internet allows for more informed choices, it also carries the risk of spreading misinformation if the sources are not credible [29]. The growing trend in online shopping appears to stem from increasing trust in digital platforms [23]. When examining product preferences, 60.4% of participants preferred domestic products, followed by French (41.7%) and German (36%) brands. These preferences align with trade data showing that Germany and France are top cosmetic exporters to Türkiye [3]. The findings suggest that brand familiarity and loyalty play a significant role in purchasing decisions.

Another important aspect of consumer behavior was the attention paid to product shelf life. The majority (85%) of participants reported checking expiration dates before purchasing cosmetics. This behavior is consistent with international studies, indicating that consumers value product safety and freshness [12,14,15,26]. The most frequently used category of cosmetic products was body care, a trend also reported in studies conducted in İzmir and Tekirdağ [3,28]. In particular, soap and basic hygiene products were found to dominate this category [27], which explains the high frequency of body care product usage. Overall, the findings of this study reflect common global trends in cosmetic use, risk perception, and consumer habits, while also emphasizing the need for improved education, access to reliable information, and professional guidance in cosmetic safety.

Cosmetovigilance awareness emerged as a critical gap in this study. In recent years, studies and reviews on cosmetovigilance have been conducted in several European Union countries; the Netherlands [16], Italy [20], France [29], Spain [30], Sweden [31] as well as in other countries, including Brazil [32], China [23], Pakistan [13], Saudi Arabia [11], the United States [33], India [12] and Japan [34]. However, studies and reviews on this topic remain quite limited in Türkiye [15,35,36,37]. Despite the relatively high educational background of the participants, 83.4% had never heard of the term “cosmetovigilance.” This finding highlights that education level alone does not guarantee awareness of consumer safety systems. A similar lack of awareness has been reported in Türkiye, where insufficient cosmetovigilance practices were documented and greater emphasis on public education was recommended [15]. International findings further support this pattern: in Brazil, only a minority of individuals experiencing adverse cosmetic effects were familiar with the NOTIVISA cosmetic safety monitoring system [32], while in China, low reporting rates underscored systemic inadequacies in cosmetovigilance [23]. Conversely, in India, although no official cosmetic safety monitoring system exists, 68.43% of participants were familiar with the concept of “cosmetic safety,” highlighting the growing recognition of its importance for public health and the need for a formalized system [12]. In contrast, the European Union (EU) has taken the lead in establishing cosmetovigilance practices, actively encouraging participation from manufacturers, healthcare professionals, and consumers [38]. Since cosmetovigilance is still an emerging field, ongoing research and awareness-raising efforts are essential to ensure the development of effective monitoring systems, particularly in developing countries where awareness remains low and reporting is insufficient [39]. While these international comparisons provide useful context regarding cosmetovigilance awareness, they should be interpreted with caution due to substantial differences in regulatory frameworks, reporting systems, and levels of institutional enforcement across countries. Moreover, variations in cosmetic product categories and market structures limit direct comparability. Therefore, these findings reflect differences in consumer awareness and reporting behaviors rather than direct equivalence between national cosmetovigilance systems.

This study has some limitations. Data were collected via self-reported questionnaires without objective verification, which may introduce reporting and recall bias. The cross-sectional design prevents determination of causality. Standardized measurement scales were not used, and social desirability may have influenced responses. Standardized measurement scales were not used; therefore, formal validity (e.g., construct validity) and reliability (e.g., Cronbach’s alpha, test–retest) assessments could not be performed. Finally, the findings may not be generalizable to populations outside Turkey.

## 5. Conclusions

This study demonstrates that despite the widespread use of cosmetics, awareness of their potential risks and cosmetovigilance remains limited in Türkiye. Improving education, encouraging professional counseling, and establishing effective monitoring systems are crucial to improving cosmetics safety and protecting public health.

## Figures and Tables

**Table 1:** Demographic characteristics of the study participants.

Variables	Descriptive Statistics
Gender	
Male	107 (15.3%)
Female	593 (84.7%)
Age	29.99 ± 10.97/25 (13–67)
Education	
Elementary or middle school	25 (3.6%)
High school	114 (16.3%)
Associate degree/university graduates	76 (10.9%)
Master’s or doctoral degree	484 (69.2%)
What types of cosmetic products do you use?	
Moisturizer	579 (82.8%)
Make-up products	418 (59.8%)
Skin care products	513 (73.4%)
Sun protection/tanning products	481 (68.8%)
Hair care/dye products	421 (60.2%)
Which country’s cosmetic products do you prefer when purchasing?	
Turkish products	423 (60.4%)
American products	143 (20.4%)
French products	293 (41.9%)
South Korean products	265 (37.9%)
German products	253 (36.1%)
Japanese products	98 (14%)
Other	43 (6.1%)
What do you use cosmetic products for?	
Protection	438 (62.7%)
Anti-aging	216 (30.9%)
Skin problems	383 (54.8%)
Beautification	336 (48.1%)

Categorical variables are shown as numbers (%). Numerical variables are shown as mean ± standard deviation/median (min–max value).

**Table 2:** Information on cosmetic toxicity and safety in Türkiye.

Variables		Descriptive Statistics
Cosmetic products are first tested on animals	Disagree	104 (14.9%)
	Agree	411 (58.7%)
	No idea	185 (26.4%)
What kind of adverse effects did you experience?	Skin redness	219 (52.1%)
	Allergy	100 (23.8%)
	Burning eyes/runny eyes	146 (34.8%)
	Itchy skin	168 (40%)
	Burning sensation	136 (32.4%)
What did you do about using cosmetic products after experiencing adverse effects?	I stopped using it	383 (79.5%)
	I changed the product brand	411 (58.7%)
	I continued using it without making any changes.	18 (3.7%)
Did you go to a doctor or a pharmacy when you experienced adverse effects?	Only to a doctor	85 (16.7%)
	Only to a pharmacy	37 (7.3%)
	Both	21 (4.1%)
	None	365 (71.9%)

Categorical variables are shown as numbers (%).

**Table 3:** Relationship between variables and knowledge of the definition of cosmetovigilance.

Variables	Have You Heard of the Term “Cosmetovigilance”?		
	No	Yes	*p* Value
**Gender** **Male** **Female**			0.078 ^b^
	96 (16.4%)	11 (9.5%)	
	488 (83.6%)	105 (90.5%)	
Where do you buy cosmetic products? Pharmacy			
	**297 (50.9%)**	**80 (69%)**	<0.001 ^a^***
Shopping malls/Cosmetic stores	385 (65.9%)	81 (69.8%)	0.416 ^a^
Online	259 (44.3%)	50 (43.1%)	0.805 ^a^
Sunscreens containing nanoparticles have harmful effects on human health			
Disagree	43 (7.4%)	25 (21.6%)	
Agree	**146 (25%)**	**49 (42.2%)**	<0.001 ^a^***
No idea	395 (67.6%)	42 (36.2%)	
Some cosmetic products can accumulate in the body			**0.001 ^a^**
Disagree	7 (1.2%%)	3 (2.6%)	
Agree	**483 (82.7%)**	**109 (94%)**	
No idea	94 (16.1%)	4 (3.4%)	
Cosmetic products are first tested on animals			
Disagree	77 (%13.2)	27 (%23.3)	
Agree	**344 (%58.9)**	**67 (%57.8)**	0.008 ^a^**
No idea	163 (%27.9)	22 (%19)	
Which country’s cosmetic products do you prefer when purchasing?			
Turkish products—yes/no (%yes)	355/229 (60.8%)	68/48 (58.6%)	0.663 ^a^
American products—yes/no (%yes)	117/467 (20%)	26/90 (22.4%)	0.649 ^b^
French products—yes/no (%yes)	**229/355 (39.2%)**	**64/52 (55.2%)**	0.001 ^a^*
South Korean products—yes/no (%yes)	**208/376 (35.6%)**	**57/59 (49.1%)**	0.006 ^a^*
German products—yes/no (%yes)	202/382 (34.6%)	51/65 (44%)	0.055 ^a^
Japanese products—yes/no (%yes)	**73/511 (12.5%)**	**25/91 (21.6%)**	0.016 ^b^*
Other—yes/no (%yes)	41/543 (7%)	2/114 (1.7%)	0.052 ^b^
Have any adverse effects occurred due to cosmetic products?—yes/no (%yes)	**268/316 (45.9%)**	**65/51 (56%)**	0.046 ^a^*
I think cosmetic products are beneficial.			
Disagree	**107 (18.3%)**	**11 (9.5%)**	0.017 ^a^*
Agree	425 (72.8%)	99 (85.3%)	
No idea	52 (8.9%)	6 (5.2%)	
Education			
Elementary or middle school	25 (4.3%)	0 (0%)	
High school	**106 (18.2%)**	**8 (6.9%)**	<0.001 ^a^***
Associate degree/university graduates	67 (11.5%)	9 (7.8%)	
Master’s or doctoral degree	385 (66%)	99 (85.3%)	

^a^: Pearson Chi-Square Test; ^b^: Yates Continuity Correction. Bold values indicate statistically significant results * *p* < 0.05; ** *p* < 0.01; *** *p* < 0.001.

**Table 4:** Correlation of the education level of cosmetic product users (*n* = 641) with variables.

Variables	Elementary/ Middle School	High School	University Graduates	Master’s/ Doctoral Degree	*p* Value
Have you heard of the term “Cosmetovigilance”? yes/no (%yes)	0/18 (0%)	8/88 (7.2%)	96/363 (20.9%)	7/61 (6.3%)	**0.001 ^a^**
Cosmetic products can accumulate in the body					
Disagree	1 (12.5%)	2 (25%)	5 (62.5%)	0 (0%)	
Agree	10 (1.8%)	78 (14.1%)	403 (72.7%)	63 (11.4%)	**0.002 ^a^**
No idea	7 (8.9%)	16 (20.3%)	51 (64.6%)	5 (6.3%)	
Herbal/organic cosmetics are natural and safe					
Disagree	3 (1.5%)	18 (8.7%)	170 (82.5%)	15 (7.3%)	
Agree	15 (4.1%)	73 (19.9%)	234 (63.9%)	44 (12%)	**<0.001 ^a^**
No idea	0 (0%)	5 (7.2%)	55 (79.7%)	9 (13%)	
Which country’s cosmetic products do you prefer?					
Turkish—yes/no (%yes)	15/3 (3.9%)	72/24 (18.9%)	254/205 (66.7%)	40/28 (10.5%)	**0.001 ^a^**
American—yes/no (%yes)	1/17 (0.7%)	21/75 (15.2%)	108/351 (78.3%)	8/60 (5.8%)	0.054 ^a^
French—yes/no (%yes)	3/15 (1.1%)	26/70 (9.3 %)	234/225 (83.3%)	18/50 (6.4%)	**<0.001 ^a^**
South Korean—yes/no (%yes)	1/17 (0.4%)	29/67 (11.1%)	208/251 (79.4%)	24/44 (9.2%)	**<0.001 ^a^**
German—yes/no (%yes)	1/17 (0.4%)	34/62 (14.1%)	192/267 (79.7%)	14/54 (5.8%)	**<0.001 ^a^**
Japanese—yes/no (%yes)	1/17 (1%)	11/85 (11.3%)	76/383 (78.4%)	9/59 (9.3%)	0.360 ^a^
Other countries—yes/no (yes %)	2/16 (5.1%)	6/90 (15.4%)	25/434 (64.1%)	6/62 (15.4%)	0.568 ^a^

^a^: Pearson Chi-Square Test. Descriptive statistics of categorical variables are presented as number (within row%).

**Table 5:** Association between Adverse Effects of Cosmetic Products and Related Variables.

Variables	Have You Experienced Any Adverse Effects Due to Cosmetic Products?		
Where do you buy cosmetic products?	**No**	**Yes**	***p* value**
Pharmacy yes/no (%yes)	170/197 (46.3%)	207/126 (62.2%)	**<0.001 ^a^**
Shopping malls/Cosmetic stores- yes/no (%yes)	226/141 (61.6%)	240/93 (72.1%)	**0.003 ^a^**
Online yes/no (%yes)	153/214 (41.7%)	156/177 (46.8%)	0.170 ^a^
Have you heard of the term “Cosmetovigilance”?			
No	316 (86.1%)	268 (80.5%)	
Yes	51 (13.9%)	65 (19.5%)	**0.046 ^a^**

^a^: Pearson Chi-Square Test. The descriptive statistics of categorical variables are presented as frequencies (within-column percentages).

## Data Availability

The original contributions presented in this study are included in the article. Further inquiries can be directed to the corresponding author(s).
